# Combined Y-shaped common channel transureteroureterostomy with Boari flap to treat bilateral long-segment ureteral strictures

**DOI:** 10.1186/1756-0500-7-550

**Published:** 2014-08-20

**Authors:** Chin-Li Chen, Shou-Hung Tang, Tai-Lung Cha, En Meng, Chih-Wei Tsao, Guang-Huan Sun, Dah-Shyong Yu, Sun-Yran Chang, Sheng-Tang Wu

**Affiliations:** Division of Urology, Department of Surgery, Tri-Service General Hospital, National Defense Medical Center, No.325, Section 2, Cheng-Kung Road, Taipei, 114 Taiwan

**Keywords:** Boari flap, Double-J catheter, Radiation, Transureteroureterostomy, Ureteral stricture

## Abstract

**Background:**

Ureteral stricture is a complication of several etiologies including idiopathic retroperitoneal fibrosis, infection, radiotherapy, instrumentation, and surgical procedures. A variety of techniques have been reported for management. The transureteroureterostomy and bladder flap have been the standard procedures for repairing distal ureteral defects of unilateral ureter. Bilateral ureteral stricture is an uncommon condition that challenges usual reconstructive procedures. It is a difficult task to reconstruct the complex situation of bilateral ureteral strictures.

**Case presentation:**

A 54-year-old female underwent concurrent chemoradiotherapy for stage IVB squamous cell carcinoma of cervix. Subsequently, she had stricture of bilateral distal ureters with bilateral hydroureteronephrosis which was found by computed tomography. The renal function deteriorated during the follow-up period. She had periodic change of double-J stents and percutaneous nephrostomy. However, the renal function still deteriorated. We performed a combined Y-shaped common channel transureteroureterostomy with Boari flap to reconstruct bilateral long-segment ureteral strictures. The patient recovered uneventfully.

**Conclusion:**

Reconstruction of bilateral ureteral strictures is a difficult treatment. We developed a modified technique for the complex situation of bilateral ureteral strictures. To our knowledge, this has not been previously reported in the scientific literature and it is a feasible procedure to treat bilateral long-segment ureteral strictures.

## Background

Ureteral strictures can result from a variety of causes, including stone passage, infection, endoscopic procedures, trauma, radiotherapy, surgery, retroperitoneal fibrosis, and malignancy. Ureteral stricture can be managed by balloon dilatation and ureteral stent initially. However, most of the strictures will recur and will require definitively surgical management. The localization, length and etiology of stricture affect the surgical modality. Short segment defects can be treated by ureteroureterostomy or ureteroneocystostomy. Management of longer ureteral defects is a potentially challenging task when the ureteral length is insufficient for direct anastomosis or reimplantation. Longer ureteral defects usually require complex procedures such as transureteroureterostomy (TUU), vesicopsoas-hitch, Boari flap, ileal ureteral substitution, or autotransplantation [1]. Bilateral ureteral stricture is an uncommon and a more challenging occurrence. We describe a case with long-segment stricture of both ureters where a complex reconstructive technique had to be employed and conventional procedures were not feasible.

## Case presentation

**Figure 1:**
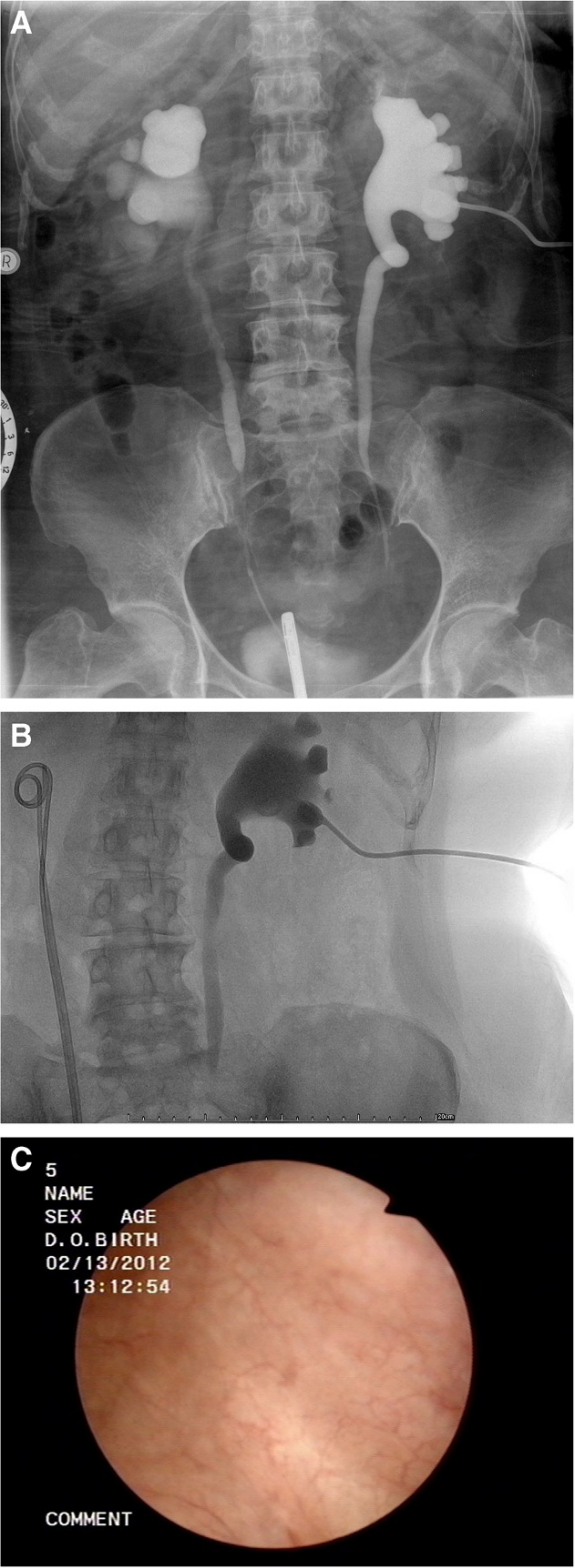
**Bilateral ureteral strictures. (A)** Retrograde pyelogram showed bilateral ureteral strictures involving middle third and lower third of ureters. **(B)** Left antegrade pyelogram showed complete obstruction at middle third of left ureter; 2 double-J catheters were inserted in right ureter. **(C)** Cystoscopy did not demonstrated obviously abnormal telangiectasia or inflammatory mucosa.

A 54-year-old female patient, with a history of stage IVB squamous cell carcinoma of cervix, was treated by concurrent chemoradiotherapy 6 years ago. Subsequently, 6 months later, bilateral hydroureteronephrosis was found by the follow-up computerized tomography (CT), which showed the stricture segment included the middle third and lower third of both ureters. The level of serum creatinine increased from 1.0 to 2.7 mg/dL. She underwent bilateral insertion of double-J catheter to facilitate the upper urinary tract drainage. Unfortunately, the follow-up CT showed persistent bilateral hydronephrosis (left side worse than right side). Additionally, the renal function deteriorated and a gradual change of serum creatinine from 2.4 to 6.5 mg/dL was found. She underwent right insertion of a parallel second double-J catheter and left percutaneous nephrostomy (Figure 1). The level of serum creatinine decreased to 2.6 mg/dL. It was possible due to the complication of retroperitoneal fibrosis and bilateral ureteral strictures following previous radiation therapy. Afterward, she had periodic change of the right side double-J stents and left percutaneous nephrostomy.

She presented with deterioration of creatinine level from 2.6 to 4.0 mg/dL and repeated catheter-related infection in recent 1 year and transferred to our hospital. Consideration of the stricture length of bilateral ureters, we planned the surgical treatment of segmental resection of bilateral ureteral stricture accompanied with bilateral Boari flap and ureteroneocystostomy.

We made a lower midline transperitoneal incision. After exposing bilateral ureters and bladder, we found a contracted bladder, generalized fibrotic change in the pelvis and bilateral long-segment ureteral strictures including middle third and lower third of ureters. We isolated the both ureters by sharp and blunt dissection and found the both dilated ureters above the junction of middle third and upper third of ureters. It was not available for the procedure of bilateral Boari flap because that the bladder was contracted and the length of bilaterally healthy ureters was not long enough, even though the bilateral upper ureters and bilateral kidneys had been mobilized.

**Figure 2:**
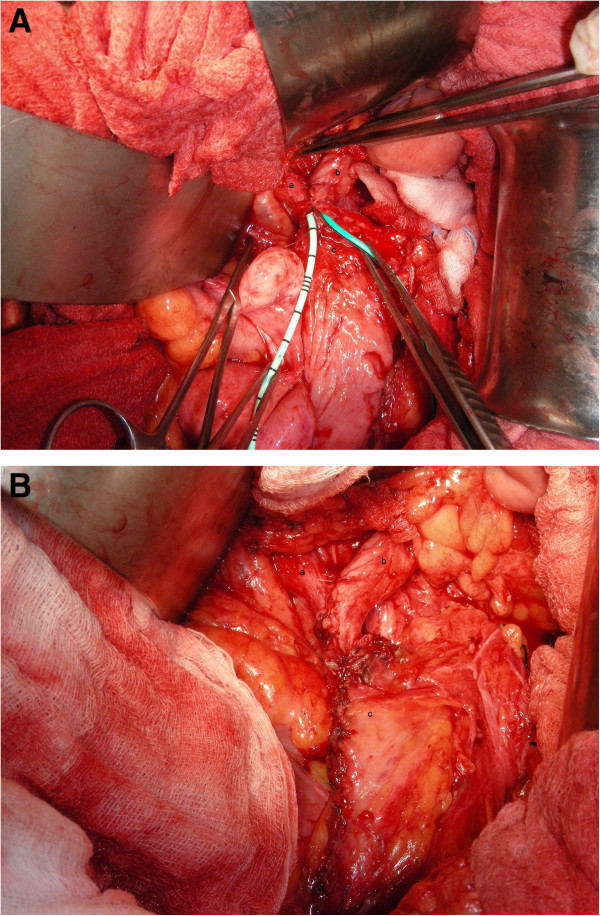
**Combined Y-shaped common channel TUU with Boari flap. (A)** and **(B)** showed the “combined Y-shaped common channel TUU with Boari flap” with bilateral double-J catheters (a) right ureter (b) left ureter (c) Boari flap.

We transected the bilateral ureters at the middle third of ureters, and placed a 3–0 synthetic absorbable stay suture in the proximal cut end of the bilateral ureters and ligated the distal stump of the bilateral ureters. After mobilizing the bladder, a psoas hitch onto the left psoas tendon was done with three interrupted sutures. We designed a Boari flap with the width 2.5 cm at the tip and 5 cm wide at the base. The right ureter was brought to the left side through the retrocolon channel. The bilateral ureters were reconstructed into a common channel, then directly anastomosed the common channel of both ureters to the edge of the Boari flap (Figure 2). Prior to closure, we inserted two double-J catheters into both ureters. We placed two closed-suction drains at the site of perivesical region and Cul-de-sac, and closed the surgical wound.

**Figure 3:**
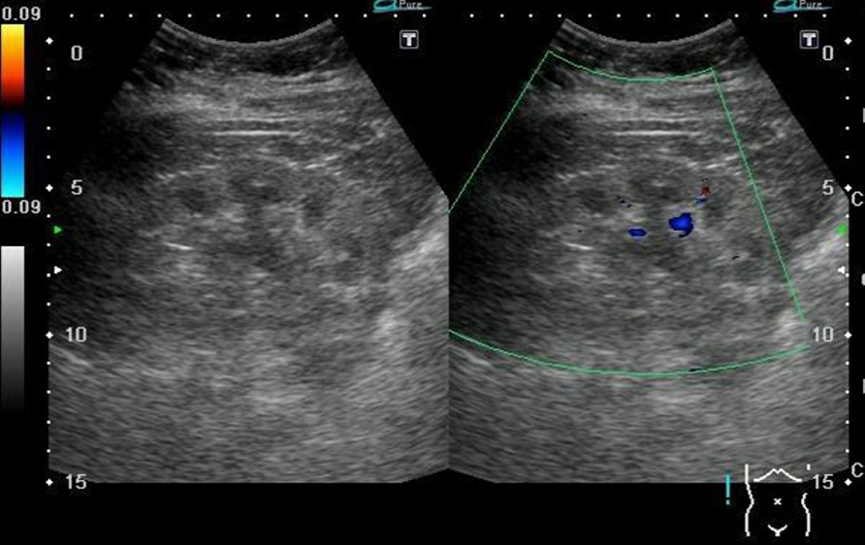
**Post-operative ultrasonography.** Ultrasonography of kidney showed mild hydronephrosis on right kidney.

After operation, she recovered uneventfully and was discharged on postoperative day 7. We removed the two double-J catheters on the fourteenth postoperative day. 18 months latter, a follow-up sonography showed mild hydronephrosis on right kidney and the level of serum creatinine was 2.4 mg/dL (Figure 3).

## Discussion

Open ureteral reconstruction is the gold standard for ureteral defects with a success rate over 90% and good long-term results [2]. In recent years, laparoscopic and robotic ureteral reconstructive surgery has been reported with good results in the literature. However, in our case, the length of ureteral stricture was long and included both ureters. Additionally, the patient underwent concurrent chemoradiotherapy before. We speculated that open ureteral reconstruction might be better in our patient because of possibility of severe retroperitoneal fibrosis.

There are various procedures for treating ureteral strictures, depending on the length, complexity and location of the lesion. Boari developed an open bladder flap operation and succeeded in an animal model in 1894 [3]. Boari flap utilizes only normal urinary tract without danger of the ipsilateral kidney nor contralateral ureter or kidney and can be done in patients with decreased renal function [4]. Thompson and Ross reported a 91% long-term success rate in patients with strictures of the lower two-thirds of the ureter [5].

TUU is used to bypass a diseased distal ureter without damaging the recipient ureter and to maintain adequate urinary drainage from the donor kidney [6]. Iwaszko reported a 96.4% long-term success rate with a patent anastomosis and a 3.6% failure rate in patients that underwent TUU for malignancy and had received radiation therapy to the pelvis before construction [7].

Radiation cystitis is a complicate problem in patients undergoing radiotherapy for gynaecological malignancies and acute condition can develop in 24-30% of patients [8]. Though most of the patients are self-limiting, it may result in chronic condition [9, 10]. Ureteral stricture is a well-known complication secondary to radiotherapy-associated fibrosis in patients with cervical carcinoma [11]. It has an incidence of 15% in patients undergoing standard doses of radiotherapy and 1% of patients developed severe ureteral fibrosis [11]. The mechanisms are not definitively understood. Some authors suggest that radiation can result in progressive endarteritis of the small blood vessels that cause cellular hypoxia and damage to fibroblasts [12]. In our opinion, the field of radiation, that extended from L4-5 interspace superiorly to 3–4 cm below the cervix and 1–2 cm lateral to the bilateral pelvic margins in our patient, also takes an important role of long-segment ureteral strictures secondary to radiotherapy-associated fibrosis.

Bilateral ureteral stricture is an uncommon occurrence and is a difficult surgical challenge. Combined use of bladder flap and TUU is an uncommon technique. Weems reported a case presented of bilateral distal ureteral stricture was successfully treated by this technique in restoring satisfactory drainage of the upper tracts and preserving use of the bladder [13]. In our case, we performed a combined Y-shaped common channel TUU with Boari flap which provided a more blunt-angle anastomosis and a larger anastomosed-lumen than the typical procedure of TUU. The technique made more smoothly urinary drainage from the both kidney and avoided the risk of ureteroureteral reflux. We predicted that lower obstruction rate and long-term success rate of the procedure.

The combination of malignancy, prior radiation and multiple prior surgeries can increase the risk of vascular compromise of the ureter and likely contribute to the rate of postoperative complications including urine leak and recurrent stricture [7]. In order to improve success rate and prevent complication of postoperative stricture, we extensively mobilized the both ureters, created a tension-free anastomosis, and preserved periureteral tissue to decrease damage of blood supply for protection from ischemic damage. And the larger common-channel of ureteral anastomosis also decreased risk of post-operative stricture. Furthermore, because of the larger common-channel, it might be possible to perform flexible ureteroscopy for further follow-up.

## Conclusions

Reconstruction of bilateral ureteral strictures is a difficult surgical challenge. To our knowledge, this is the first reported case to use a “combined Y-shaped common channel TUU with Boari flap” for reconstruction of bilateral long-segment ureteral strictures involving middle third and lower third of ureter. This technique provides a good tension-free repair over the anastomosis. In conclusion, it is an efficacious and feasible procedure for long-segment strictures of both ureters.

## Consent

Written informed consent was obtained from the patient for publication of this case report and any accompanying images. A copy of the written consent is available for review by the Editor of this journal.

## References

[1] Rassweiler JJ, Gozen AS, Erdogru T, Sugiono M, Teber D (2007). Ureteral reimplantation for management of ureteral strictures: a retrospective comparison of laparoscopic and open techniques. Eur Urol.

[2] Gozen AS, Cresswell J, Canda AE, Ganta S, Rassweiler J, Teber D (2010). Laparoscopic ureteral reimplantation: prospective evaluation of medium-term results and current developments. World J Urol.

[3] Boari A (1894). Contribute sperementale alla plastica delle uretere. Atti Accad Med Ferrara.

[4] Olsson CA, Norlen LJ (1986). Combined Boari bladder flap-psoas bladder hitch procedure in ureteral replacement. Scand J Urol Nephrol.

[5] Thompson IM, Ross G (1974). Long-term results of bladder flap repair of ureteral injuries. J Urol.

[6] Barry JM (2005). Surgical atlas transureteroureterostomy. BJU Int.

[7] Iwaszko MR, Krambeck AE, Chow GK, Gettman MT (2010). Transureteroureterostomy revisited: long-term surgical outcomes. J Urol.

[8] Shakespeare TP, Lim KH, Lee KM, Back MF, Mukherjee R, Lu JD (2006). Phase II study of the American Brachytherapy Society guidelines for the use of high-dose rate brachytherapy in the treatment of cervical carcinoma: is 45–50.4 Gy radiochemotherapy plus 31.8 Gy in six fractions high-dose rate brachytherapy tolerable?. Int J Gynecol Cancer.

[9] Pavlidakey PG, MacLennan GT (2009). Radiation cystitis. J Urol.

[10] Maduro JH, Pras E, Willemse PH, de Vries EG (2003). Acute and long-term toxicity following radiotherapy alone or in combination with chemotherapy for locally advanced cervical cancer. Cancer Treat Rev.

[11] Buglione M, Toninelli M, Pietta N, Ambrosi E, Filippini M, De Stefani A, Vitali E, De Tomasi D, Bertoni F, Caraffini B, Magrini SM (2002). [Post-radiation pelvic disease and ureteral stenosis: physiopathology and evolution in the patient treated for cervical carcinoma. Review of the literature and experience of the Radium Institute]. Arch Ital Urol Androl.

[12] Craighead P, Shea-Budgell MA, Nation J, Esmail R, Evans AW, Parliament M, Oliver TK, Hagen NA (2011). Hyperbaric oxygen therapy for late radiation tissue injury in gynecologic malignancies. Curr Oncol (Toronto, Ont).

[13] Weems WL (1970). Combined use of bladder flap and transureteroureterostomy: report of a case. J Urol.

